# Sex-specific effects of gastrointestinal microbiome disruptions on *Helicobacter pylori*-induced gastric carcinogenesis in INS-GAS mice

**DOI:** 10.1186/s13293-025-00700-z

**Published:** 2025-02-21

**Authors:** Chao Peng, Xin Li, Yu Li, Xinbo Xu, Yaobin Ouyang, Nianshuang Li, Nonghua Lu, Yin Zhu, Cong He

**Affiliations:** 1 Department of Gastroenterology, Jiangxi Provincial Key Laboratory of Digestive Diseases, Jiangxi Clinical Research Center for Gastroenterology, Digestive Disease Hospital, The First Affiliated Hospital, Jiangxi Medical College, Nanchang University, 17 Yong Waizheng Street, Donghu District, Nanchang, 330006 Jiangxi China; 2 HuanKui Academy, Nanchang University, Nanchang, China

**Keywords:** Sex difference, Gastric cancer, *Helicobacter pylori*, Gastrointestinal microbiota, 16S rRNA gene sequencing

## Abstract

**Background:**

Accumulating evidence indicates that the dysbiosis of gastrointestinal microbiota is associated with the development of gastric carcinogenesis. However, the sex-specific traits of gastrointestinal microbiota and their correlation with the sexually dimorphic response to gastric cancer remain poorly understood.

**Methods:**

Male and female transgenic FVB/N insulin-gastrin (INS-GAS) mice as a model of gastric cancer were randomly administered Brucella Broth or *Helicobacter pylori* (*H. pylori*). Stomachs were evaluated by histopathology. The gastric inflammation was examined by immunohistochemical and immunofluorescence staining. Gastric mucosal and fecal samples were collected for microbiota analysis using 16S rRNA gene sequencing.

**Results:**

Following *H. pylori* infection, male mice showed heightened inflammatory infiltration and notably greater intestinal metaplasia compared to female mice. The structure of gastrointestinal microbiota was different between male and female mice, with relative higher diversity in females than males. Notably, we found gender disparities in the alterations of gastric and intestinal microbiota in mice post *H. pylori* infection. While the enrichment of *Bifidobacterium* and *Lachnospiraceae* was observed in female mice, *Escherichia_Shigella* and *Akkermansia* were more abundant in males. Furthermore, the microbial profile was distinct in estrogen-deficient ovariectomized (OVX) mice, including the overgrowth of *Akkermansia* and the loss of *Butyricicoccus*. Infected OVX females developed significantly more severe gastric lesions, which was normalized through co-housing with intact females.

**Conclusions:**

We have identified a novel microbiome-based mechanism that provides insight into the sexual dimorphism in the development of *H. pylori*-associated gastric cancer.

**Supplementary Information:**

The online version contains supplementary material available at 10.1186/s13293-025-00700-z.

## Plain language summary

Men have a higher incidence of gastric cancer and a poorer prognosis compared to women, yet the underlying mechanisms remain unclear. Recent evidence suggests that gastrointestinal microbiota, in addition to *H. pylori*, play a critical role in the progression of gastric carcinogenesis. This study utilized the *H. pylori* infected gastric cancer model INS-GAS mice to investigate the gastric pathology in different genders and the subsequent changes in their gastrointestinal microbiota. We observed that the gastric lesions, including inflammation and precancerosis, were more pronounced in male mice than in females. Interestingly, the composition of gastrointestinal microbiota was different between males and females. While males displayed an enrichment of pathogens, such as *Escherichia_Shigella* and *Streptococcus*, females exhibited an over-representation of the beneficial *Bifidobacterium*. To further explore the role of estrogen, we constructed ovariectomized mice. The results showed that the gastric injury was exacerbated after estrogen deprivation. The structure of gut microbiota was distinct between ovariectomized and intact mice. Considering the coprophagy behavior of mice, we housed ovariectomized and intact mice together to examine the effect of exchanging gut microbiota on gastric pathology. The results revealed that the disparity in gastric lesions between the two groups was weakened after co-housing experiment. Our findings suggest that the gastrointestinal microbiota may act as a mediator in the protective role of estrogen against gastric carcinogenesis. Therefore, elucidating the role of the gastrointestinal microbiota in the initiation and progression of gastric cancer will offer novel perspectives and a foundational framework for gastric cancer prevention strategies.

**Supplementary Information:**

The online version contains supplementary material available at 10.1186/s13293-025-00700-z.

## Highlights

1. The gastric lesions were more severe in male mice compared to females after *H. pylori* infection.
2. The infection of *H. pylori* induced alterations of both gastric and gut microbiota, including the enrichment of *Helicobacter* and the depletion of short chain fatty acids-producing bacteria.
3. The response of gastrointestinal microbiota to *H. pylori* infection differed between genders, with females exhibiting a higher abundance of *Bifidobacteria* than males.
4. The deprivation of estrogen aggravated gastric lesions caused by *H. pylori* infection, along with the dysbiosis of gut microbiota.
5. Co-housing ovariectomized mice with intact females neutralized the disparity in gastric injury.

**Supplementary Information:**

The online version contains supplementary material available at 10.1186/s13293-025-00700-z.

## Introduction

Gastric cancer (GC) is the fifth leading cause of cancer-related mortality worldwide, accounting for more than 659,853 deaths in 2022 [1]. In general, it is believed that GC develops via a predictable progression from superficial gastritis, atrophic gastritis, intestinal metaplasia, and subsequently to cancer. This multistep progression cascade is often initiated by *Helicobacter pylori* (*H. pylori*) infection, which has been recognized as the strongest risk factor for GC [2]. Interestingly, men present with larger, higher stage, higher grade GC as well as poor outcome than women [3]. Although GC incidence varies among geographical regions, men are two times more likely than women to develop the disease in both high and low risk areas [4]. Another in vivo study indicated that female mice displayed lower risk of GC, and ovariectomy (OVX) increased the severity of gastric mucosal lesions. The degree of pathological changes and incidence of GC was reduced after supplementing estrogen, demonstrating that sex hormones modulate GC risk[5]. Nevertheless, the underlying mechanism of sex dimorphism in GC is unclear.

Apart from *H. pylori*, there is growing evidence that non-*H. pylori* microbiota in the stomach plays a role in the development of GC, originating primarily from the oral cavity and gut[6]. Studies have reported that patients with GC exhibited significant alterations in gut microbial structure compared to healthy controls, indicating that these changes could serve as non-invasive, accurate and sensitive markers for early GC diagnosis[7, 8]. The crucial effect of gut microbiota in GC has also been confirmed in germ-free transgenic FVB/N insulin-gastrin (INS-GAS) mice, which displayed lower degree of gastric mucosal lesions and prolonged carcinogenesis time[9]. Apart from environmental exposures (including diet and medication), host factors such as genetics and hormones are linked to variations of gut microbiota[10]. Moreover, gender-specific gut microbiota have been associated with the sexual dimorphism seen in several diseases, including type 2 diabetes and cardiometabolic dysfunction[11]. However, the sex-specific effects of gastrointestinal microbiota in the development of GC are not well understood.

In this study, we validated that the *H. pylori*-associated gastric lesions were more severe in male mice compared to females. We identified disparities in the structure of gastric and gut microbiota between male and female mice, with females exhibiting higher diversity than males. The alterations of gastrointestinal microbiota in male mice differed from those in females in response to *H. pylori* infection. Additionally, a higher abundance of *Bifidobacterium* was observed in both the stomach and intestine of infected female mice compared to males. We also found that estrogen deprivation worsened gastric injury induced by *H. pylori* infection, accompanied by microbial disturbance. Co-housing ovariectomized (OVX) mice with intact counterparts normalized their pathological differences. Our findings offer valuable insights into understanding the interactions between gender and microbiota in determining the susceptibility of GC.

## Materials and methods

### Bacterial strains and cultivation conditions

The rodent-adapted CagA + *H. pylori* strain pre-murine Sydney Strain 1 (PMSS1) was used in this study. The bacteria were cultured on Brucella agar (BD, Biosciences) with 5% sheep blood (BD Bioscience) and incubated at 37 °C under microaerophilic conditions. The bacterial density was detected by spectrophotometry at 600 nm.

### Animal experiment

Six to eight-week 30 male and 52 female specific-pathogen-free INS-GAS mice (Jackson Laboratory) were kept with a 12 h light/dark cycle and provided ad libitum access to food and water. The air conditions were controlled at a temperature of 18–26 °C with 40%–70% relative humidity. The male and female mice were divided into three groups with 2–3 mouse per cage in each treatment group, including non-*H. pylori*-infected, *H. pylori*-infected for 4 months, and *H. pylori*-infected for 7 months groups. INS-GAS mice were gavaged with Brucella Broth or challenged with 2 × 10^9^ colony forming units (CFU)/mouse *H. pylori* strain once every other day for five times. Ovariectomy and sham surgeries were conducted at 8 weeks of age. Two weeks after the surgeries, mice were either infected with *H. pylori* or administered broth only. To normalize the gut microbiome, OVX mice were co-housed together with intact females for 7 months. All procedures performed on animals were approved by the Ethics Committee of First Affiliated Hospital of Nanchang University.

### Sample collection and histological analysis

The mice were allowed to defecate naturally under sterile conditions and the feces were collected using sterile forceps. During the collection process, we utilized alcohol to sterilize the forceps in order to prevent cross-contamination between individual mice. A minimum of 3 fecal pellets were collected from each mouse, and aliquoted into 2ml sterile centrifuge tubes. After collection, the samples were rapidly frozen in liquid nitrogen for 15 min before being transferred to storage at -80℃.

Mice were fasted overnight before necropsy. For histopathologic evaluation, the stomach was incised along the greater curvature. Luminal contents were removed, and the mucosa was rinsed with sterile saline. Then, linear strips extending from squamocolumnar to proximal duodenum were taken along the lesser curvature, fixed overnight in 10% neutral-buffered formalin, embedded in paraffin, cut into 4 μm-thick sections for hematoxylin and eosin (H&E) for histopathology. Inflammation, oxyntic gland atrophy, foveolar hyperplasia, intestinal metaplasia, and dysplasia in the gastric cardia and corpus were scored from 0 to 4 according to previously published criteria[12]. *H. pylori* infection was confirmed by Giemsa staining[13].

#### Immunohistochemical staining

Immunohistochemistry analysis was conducted to evaluate the infiltration of inflammatory cells in gastric tissues, as described previously[14]. In brief, tissue sections were deparaffinized in a 70 ℃ oven for 1.5 h, followed by rehydration using a graded ethanol series. Antigen retrieval was carried out by boiling the sections in citrate buffer in a microwave for 15 min. Endogenous peroxidase activity was quenched by incubating in 3% H_2_O_2_ for 10 min. The sections were then incubated overnight at 4 ℃ with the primary antibodies (anti-myeloperoxidase rabbit polyclonal antibody, ab9535, Abcam, UK; anti-F4/80 antibody, GB11027, Servicebio, China; anti-IL-1β, D4T2D, Cell Signaling Technology, USA). Subsequently, tissue sections were treated with the secondary antibody (PV-6000, Zhongshan Biotech, China) and visualized using a DAB staining kit following the manufacturer’s instructions (Zhongshan Biotech, China). The quantitative analysis of immunohistochemistry staining was performed as previously described[15]. The positive cells were calculated by the staining intensity scores (0 (normal), 1 (weak), 2 (medium), 3 (strong)) and staining area (0: 0%, 1:1%-25%, 2: 26%-50%, 3: 51%-75%, 4: 76%-100%).

#### Immunofluorescence staining

The tissue sections underwent deparaffinization and rehydration steps, followed by antigen retrieval in citrate buffer (pH 6.0). Subsequently, the sections were permeabilized with 0.3% Triton X-100 in PBS for 15 min and then blocked with 3% BSA in PBS for 1 h. The anti-TNF-α antibody (SC-52746, Santa Cruz, USA) was applied overnight at 4 °C. For immunofluorescence staining, the tissue slides were rinsed with PBS and incubated with an Alexa-flour secondary antibody (A-11001, Invitrogen, USA) at room temperature for 60 min, followed by mounting with a suitable medium containing 4′,6-diamidino-2-phenylindole (DAPI) nuclear counterstain. The mounted sections were then imaged using a confocal microscope (LEICA Stellaris 5).

### DNA extraction and amplification

Total bacterial DNA was extracted from gastric mucosal and fecal samples using the FastPure Stool DNA isolation Kit (MJYH, China) according to the instructions of the manufacturer. The quality and concentration of DNA were detected using NanoDrop2000 (Thermo Fisher Scientific, USA). The DNA integrity was evaluated by 1% agarose gel electrophoresis. The hypervariable V3–V4 regions of the 16S rRNA gene were amplified using primers 338F (5′-ACTCCTACGGGAGGCAGCAG-3′) and 806R (5′-GGACTACHVGGGTWTCTAAT-3′) with a T100 Thermal Cycle (BIO-RAD, USA). The PCR reaction mixture included 4 μL of 5 × FastPfu buffer, 2 μL of 2.5 mM dNTPs, 0.8 μL of 5 μM for each primer, 0.4 μL of FastPfu polymerase, 10 ng of template DNA, and ddH_2_O to a final volume of 20 μL. The PCR amplification cycling conditions were as follows: initial denaturation at 95 °C for 3 min, followed by 27 cycles of denaturing at 95 °C for 30 s, annealing at 55 °C for 30s, and extension at 72 °C for 45 s, with a final extension at 72 ℃ for 10 min, and ending at 4 °C. All samples were amplified in triplicate. The PCR product was extracted from a 2% agarose gel, purified and quantified using Synergy HTX (Biotek, USA).

### Library construction and sequencing

Purified amplicons were pooled in equimolar quantities, and a sequencing library was constructed according to the official details of the Illumina. Subsequently, next-generation sequencing was performed using an Illumina NextSeq 2000 PE300 platform (Illumina, USA) by Majorbio Bio-Pharm Technology Co. Ltd. with paired-end reads.

### Bioinformatic analysis

Raw sequencing reads were demultiplexed, quality-filtered by FASTP version 0.20.0 and merged by FLASH version 1.2.7[16, 17]. The specified criteria were as follows: (i) Reads were truncated at sites receiving an average quality score of < 20 over a 50 bp sliding window, and reads shorter than 50 bp after truncation, as well as those containing ambiguous characters, were removed; (ii) Assembly included only overlapping sequences longer than 10 bp based on their overlapping regions, allowing for a maximum mismatch ratio of 0.2. Reads that could not be assembled were eliminated; (iii) Samples were delineated by barcode and primers, adjusting the sequence orientation with precise barcode matching and permitting a 2-nucleotide mismatch in primer alignment. Operational taxonomic units (OTUs) with 97% similarity cutoff were clustered using UPARSE version 7.1 and chimeric sequences were removed[18]. The taxonomy of each OTU representative sequences was analyzed by RDP Classifier version 2.2 against Silva 16S rRNA database (Version 138) using confidence threshold of 0.7[19]. The microbial diversity in samples was estimated using the alpha diversity that includes Chao and Shannon index. Alpha diversity estimator calculations were performed using Mothur version 1.30.2 based on OTU level. Principal coordinates analysis (PCoA) based on Bray–Curtis distance were calculated using R (version 3.3.1) to assess beta diversity. Significant differences of the beta diversity among groups were estimated by Adonis test using the vegan package in R (version 3.3.1). The bacterial taxonomic distributions of each group were visualized using R (version 3.3.1). The specific characterization of microbiota that differentiate among groups was evaluated using a linear discriminant analysis (LDA) effect size (LEfSe) algorithm.

### Statistical analysis

The data were analyzed using SPSS 26.0 for Windows (SPSS Inc., USA) and presented as means and standard error of the mean (SEM). Differences between two groups following a normal distribution were assessed using the Student’s t-test, while comparisons among more than two groups were conducted through one-way analysis of variance. Post hoc analysis with the least significant difference (LSD) test was performed when ANOVA yielded significant results. For groups without a normal distribution, differences were evaluated using the Mann–Whitney *U* test for two groups and the Kruskal–Wallis *H* test for more than two groups. The Mann–Whitney *U* test served as the post hoc test upon significance of the Kruskal–Wallis *H* test. Statistical significance was considered when p values were less than 0.05. N represented the number of samples in each analysis.

## Results

### Gender heterogeneity in *H. pylori*-induced gastric lesions

**Fig. 1:**
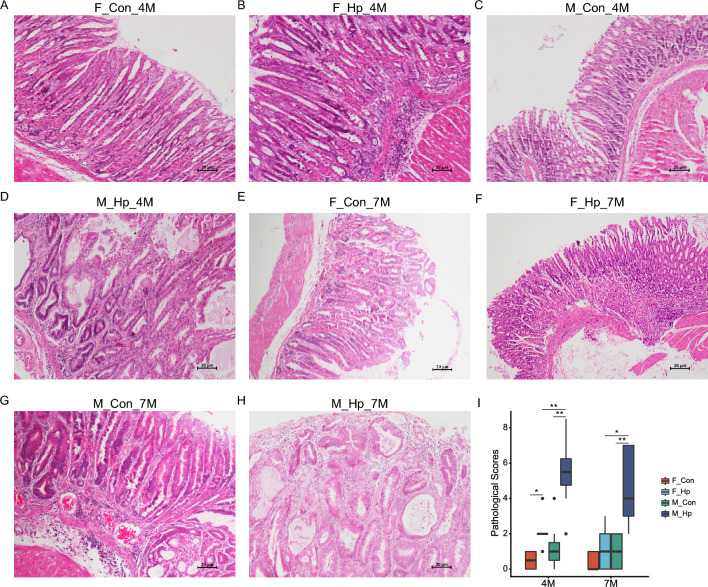
Histopathology of the stomach in male and female INS-GAS mice following 4 and 7 months of *H. pylori* infection. Uninfected (**A**) and infected (**B**) females at 4 months; Uninfected (**C**) and infected (**D**) males at 4 months; Uninfected (**E**) and infected (**F**) females at 7 months; Uninfected (**G**) and infected (**H**) males at 7 months. Scale bar: 20 μm. **I** Histological scores. n = 6–9/group. N represented the number of mice. *p < 0.05. **p < 0.01. ***p < 0.001. F_Con, females without infection. F_Hp, females with infection. M_Con, males without infection. M_Hp, males with infection

**Fig. 2:**
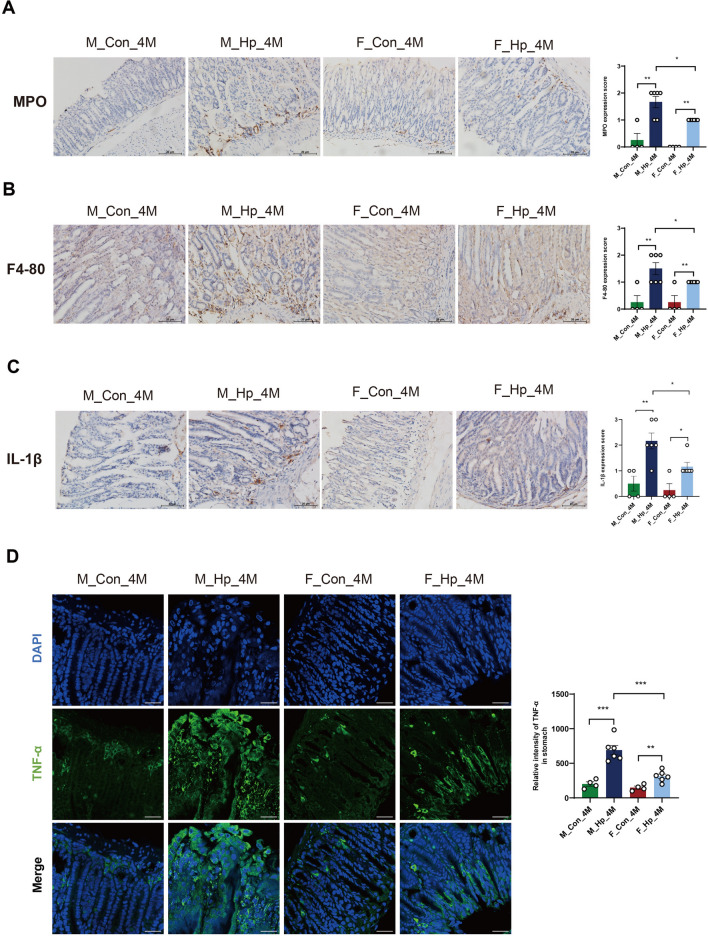
The gastric inflammation after 4 months of *H. pylori* infection. Myeloperoxidase (MPO) (**A**), F4/80 (**B**), IL-1β (**C**) immunohistochemistry of gastric tissues from males and females with or without *H. pylori* infection. Scale bar: 20 μm. (**D**) Gastric staining of TNF-α (green) and DAPI (blue) to visualize nuclei by Immunofluorescene (× 600). n = 4–6/group. N represented the number of mice. *p < 0.05. **p < 0.01. ***p < 0.001. M_Con_4M, males without infection at 4 months. M_Hp_4M, males following 4 months infection. F_Con_4M, females without infection at 4 months. F_Hp_4M, females following 4 months infection

**Fig. 3:**
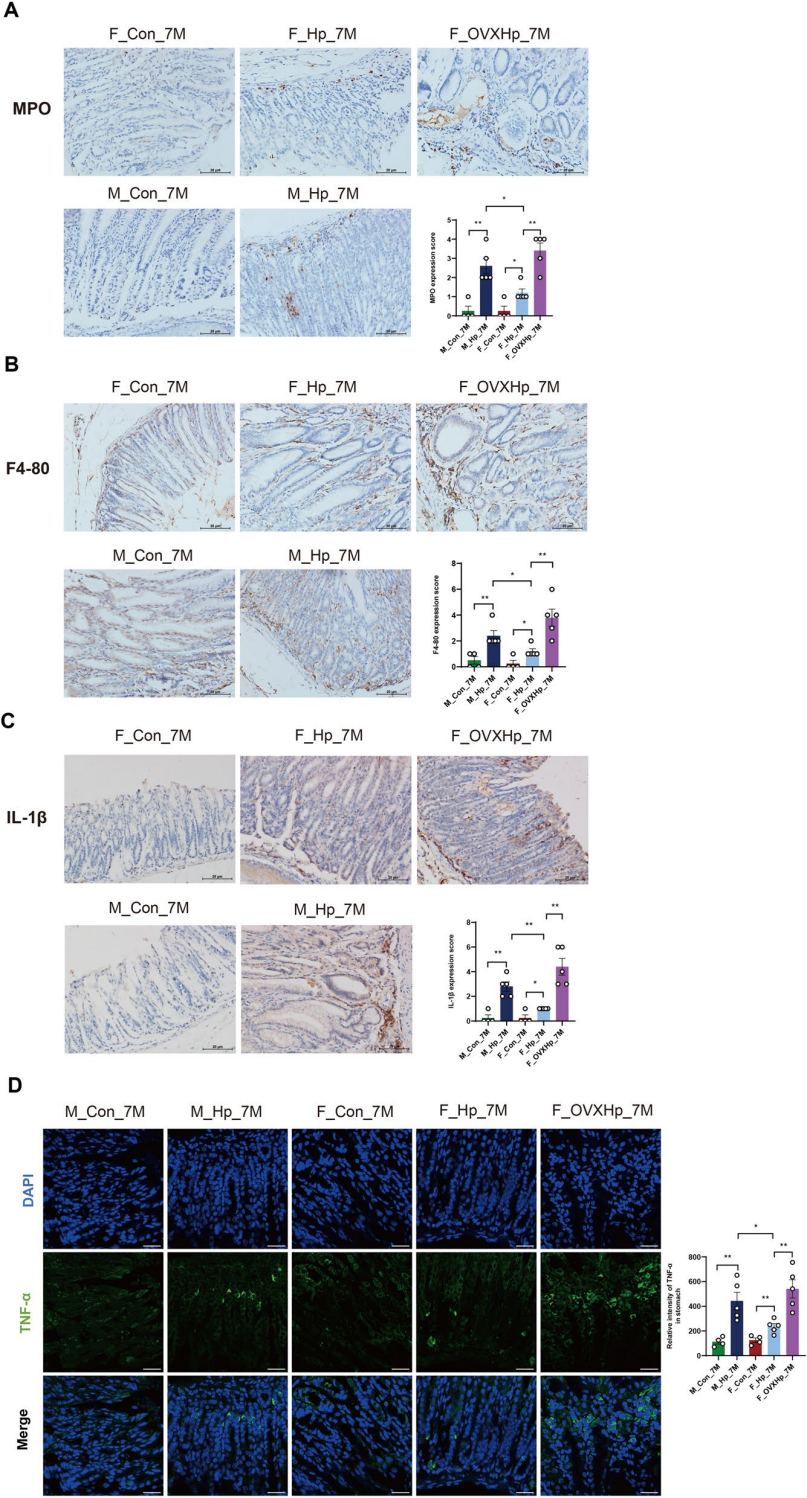
The gastric inflammation after 7 months of *H. pylori* infection. Myeloperoxidase (MPO) (**A**), F4/80 (**B**), and IL-1β (**C**) immunohistochemistry of gastric tissues from males, females, and ovariectomized females. Scale bar: 20 μm. **D** Gastric staining of TNF-α (green) and DAPI (blue) to visualize nuclei by Immunofluorescene (× 600). n = 4–5/group. N represented the number of mice. *p < 0.05. **p < 0.01. M_Con_7M, males without infection at 7 months. M_Hp_7M, males following 7 months infection. F_Con_7M, females without infection at 7 months. F_Hp_7M, females following 7 months infection. F_OVXHp_7M, ovariectomized females following 7 months infection

The successful infection of *H. pylori* has been confirmed through Giemsa staining (Additional file 1: figure S1). As depicted in Fig. 1, the median histological scores were higher in *H. pylori*-infected mice compared to those in uninfected mice after 4 months. There were no significant differences of histological parameters between males and females in the absence of infection (Fig. 1A, C). Of note, infected male mice displayed evident inflammatory cell infiltration and intestinal metaplasia in the gastric mucosa, whereas infected female mice exhibited only mild inflammatory responses and minimal occurrence of metaplasia (Fig. 1B, D). At 7 months post-administration, uninfected male mice showed more severe gastritis compared to females, with some males developing intestinal metaplasia, while females displayed no signs of metaplasia (Fig. 1E, G). Likewise, male mice demonstrated a higher frequency of intestinal metaplasia and a more advanced grade of dysplasia than female mice following long-term *H. pylori* infection (Fig. 1F, 1H). Additionally, we found a significantly elevated expression of MPO, F4/80, TNFα and IL-1β in the stomachs of male mice compared to female mice, both 4 months and 7 months after *H. pylori* infection (Figs. 2 and 3). Taken together, the progression of GC showed gender dimorphism, with aging male mice spontaneously developing lesions, accelerated by *H. pylori* infection, while females exhibited milder manifestations.

### Sex-specific characterization of gastric microbiota after *H. pylori* infection

**Fig. 4:**
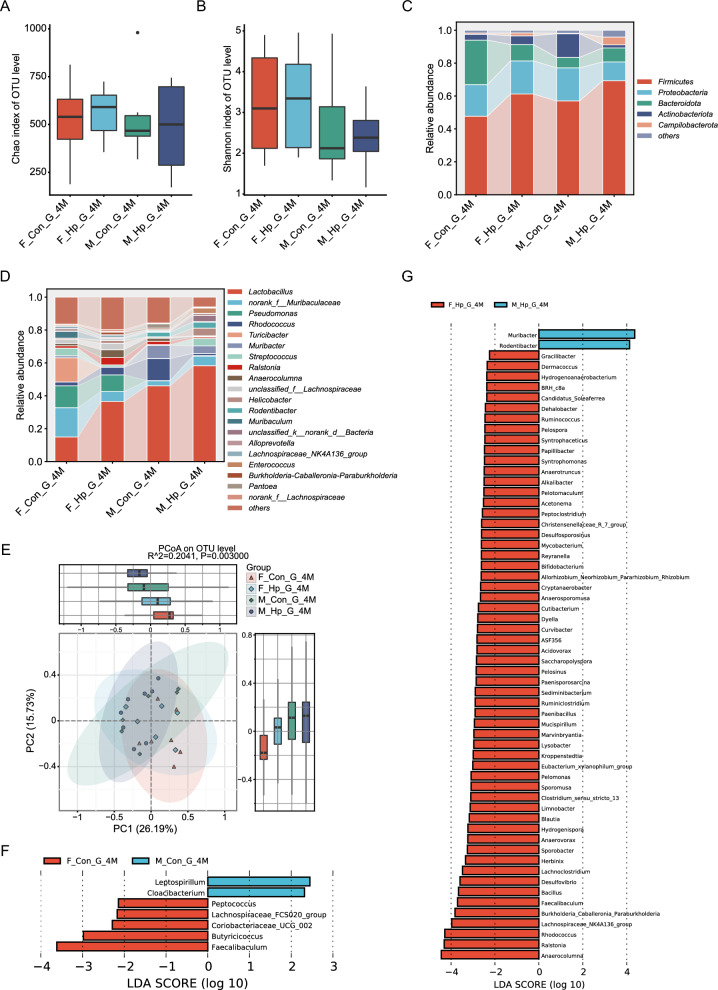
Alterations of gastric microbiota in male and female mice following 4 months of *H. pylori* infection. Alpha diversity indices, including Chao (**A**) and Shannon (**B**). Relative abundance of bacterial phyla (**C**) and genus (**D**). **E** Principal coordinate analysis (PCoA) of the Bray–Curtis distances. The linear discriminant analysis (LDA) effect size (LEfSe) was conducted to identify the differentially abundant bacteria among male and female with (**G**) and without (**F**) infection. n = 6–8/group. N represented the number of mice. F_Con_G_4M, gastric samples from females without infection at 4 months. F_Hp_G_4M, gastric samples from females following 4 months infection. M_Con_G_4M, gastric samples from males without infection at 4 months. M_Hp_G_4M, gastric samples from males following 4 months infection

**Fig. 5:**
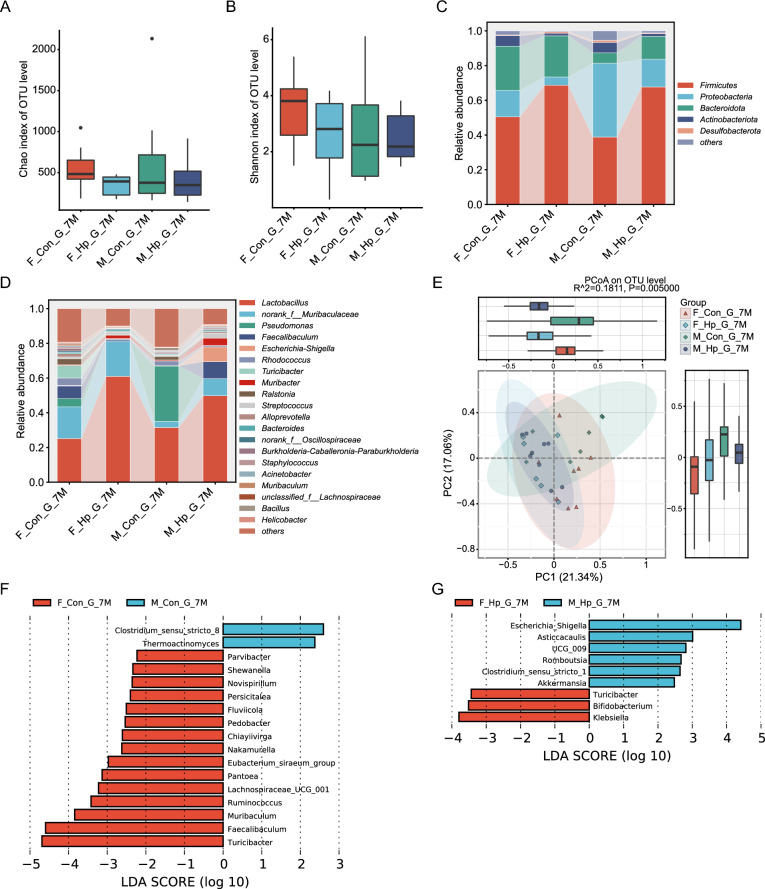
Changes in gastric microbiota in male and female mice after 7 months of *H. pylori* infection. Alpha diversity of gastric microbiota measured by Chao (**A**) and Shannon (**B**) indexes. Bar plots showing the mean relative abundances of the top 5 phyla (**C**) and 20 genera (**D**). **E** PCoA plot based on OTU-level Bray–Curtis dissimilarity showing the separation of gastric samples. LEfSe analysis was conducted to identify the bacteria with differential abundance between males and females with (**G**) and without (**F**) infection. F_Con_G_7M, gastric samples from females without infection at 7 months. n = 6–9/group. N represented the number of mice. F_Hp_G_7M, gastric samples from females following 7 months infection. M_Con_G_7M, gastric samples from males without infection at 7 months. M_Hp_G_7M, gastric samples from males following 7 months infection

16S rRNA gene sequencing of gastric mucosa samples demonstrated that the Chao index, reflecting microbial richness, and the Shannon index, reflecting microbial diversity, were not significantly different following *H. pylori* infection, neither at 4 months (Fig. 4A–B) nor at 7 months (Fig. 5A–B). Female mice exhibited a tendency towards a higher Shannon index compared to males. Beta diversity was calculated using Bray–Curtis distance visualized in PCoA plots, which could display the similarities and differences between samples and reflect within-group variability through the distribution of sample points (Fig. 4E, 5E). Overall, the gastric microbiota composition of female mice was distinctly different from that of male mice regardless of *H. pylori* infection (Adonis test, R^2^ = 0.0458, p = 0.005). The infected group displayed a distinct microbial structure compared to the uninfected mice (Adonis test for 4 months, R^2^ = 0.2041, p = 0.003; Adonis test for 7 months, R^2^ = 0.1811, p = 0.005).

In the stomach, Firmicutes, Proteobacteria, and Bacteroidota emerged as the top three most abundant phyla, showcasing distinct distributions between males and females in the presence or absence of *H. pylori* infection (Fig. 4C, 5C). In the infected mice, there was a notable increase in the relative abundance of Firmicutes compared to the uninfected group, while Actinobacteriota showed a decrease. At the genus level, *Lactobacillus*, a member of the Firmicutes phylum, was prevalent in male mice following 4 months of *H. pylori* infection (Fig. 4D, 5D). Additionally, higher abundances of *Helicobacter*, *Streptococcus*, *Enterococcus* were observed in the infected group compared to the uninfected mice (Additional file 2: figure S2F).

In the absence of *H. pylori* infection, male mice demonstrated a decreased abundance of *Butyricicoccus, Faecalibaculum,* and *Ruminococcus,* along with an increased abundance of *Leptospirillum,* and *Cloacibacterium* compared to female mice (Fig. 4F, 5F). The bacterial differences between male and female mice were more pronounced after *H. pylori* infection, particularly at 4 months. While infected male mice exhibited enrichment of *Muribacter, Escherichia-Shigella,* and *Akkermansia*, females with *H. pylori* infection were primarily characterized by higher levels of *Blautis*, *Lachnoclostridium*, and *Bifidobacterium* (Fig. 4G, 5G). Of note, the over-representation of beneficial *Bifidobacterium* in female mice compared to males was consistently present at 4 months and 7 months post infection. As the duration of *H. pylori* infection extended, there were distinct changes in the composition of gastric microbiota between male and female mice (Additional file 3: figure S3B, S3D). After 7 months of infection, male mice showed a significant increase in the relative abundance of *Escherichia-Shigella*, whereas female mice exhibited a decrease (Fig. 5G). Taken together, these results suggest that the gastric microbiota in male and female mice respond differently to *H. pylori* infection.

### Sexual dimorphism of gut microbiota and the alterations post *H. pylori* infection

**Fig. 6:**
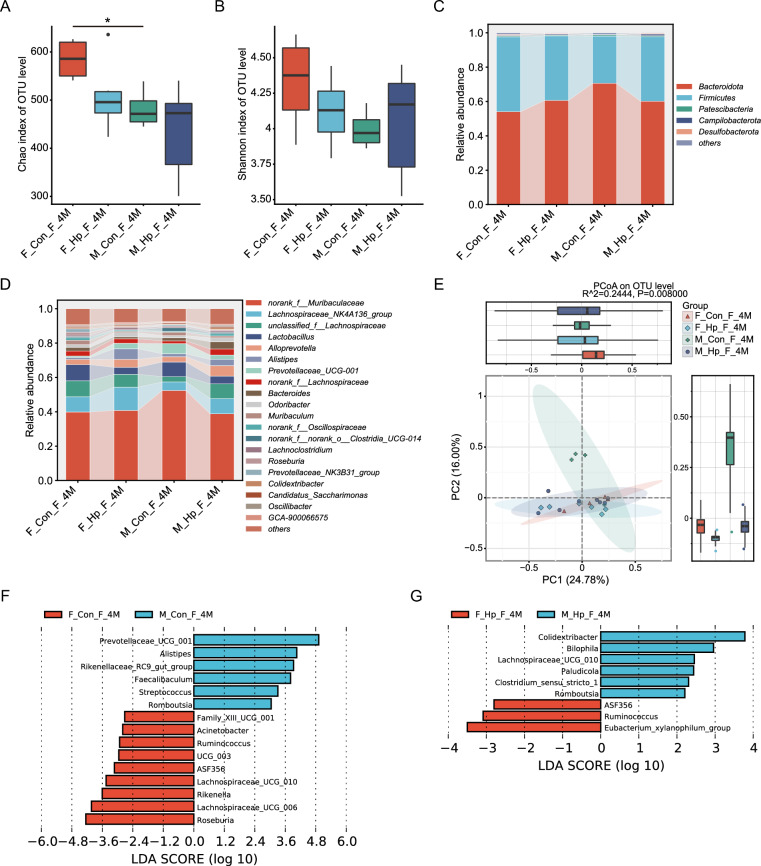
Effects of *H. pylori* infection on gut microbiota in mice of different genders after 4 months. Alpha diversity of gut microbiome, as indicated by Chao (**A**) and Shannon (**B**) indices. The Relative abundance of the dominant phyla (**C**) and genera (**D**). **E** PCoA of the fecal samples from mice with and without infection. LEfSe analysis of discriminant genera of gut microbiome between males and females with (**G**) and without (**F**) infection. n = 4–8/group. N represented the number of samples. *, *p* < 0.05. F_Con_F_4M, fecal samples from females without infection at 4 months. F_Hp_F_4M, fecal samples from females following 4 months infection. M_Con_F_4M, fecal samples from males without infection at 4 months. M_Hp_F_4M, fecal samples from males following 4 months infection

**Fig. 7:**
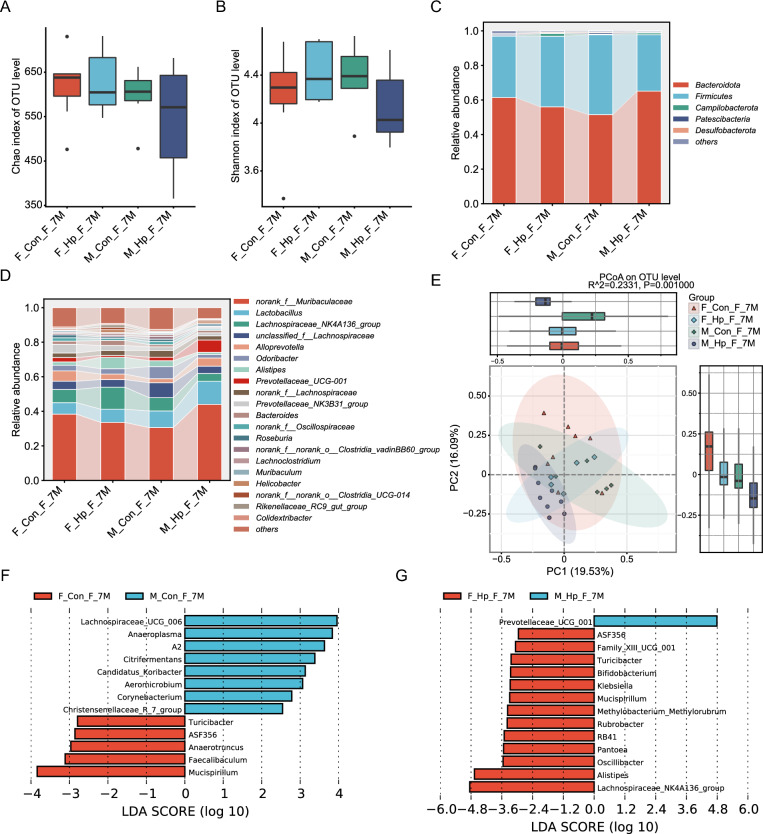
Gender disparities in gut microbiota alterations following 7 months of *H. pylori* infection. Chao (**A**) and Shannon (**B**) diversity indices and PCoA (**E**) score plots of the gut microbiota with and without infection in male and female mice. Average relative abundance of top five phyla (**C**) and top 20 genera (**D**) detected in stools from mice. **F**, **G** Linear discriminant analysis effect size in gut microbiome of mice after 7 months of administration. n = 5–9/group. N represented the number of samples. F_Con_F_7M, fecal samples from females without infection at 7 months. F_Hp_F_7M, fecal samples from females following 7 months infection. M_Con_F_7M, fecal samples from males without infection at 7 months. M_Hp_F_7M, fecal samples from males following 7 months infection

Analysis of microbial alpha diversity in fecal samples revealed that the Chao and Shannon indexes were lower in male controls than in females at 4 months (Fig. 6A-B). There were no significant differences in alpha diversity indexes observed between the *H. pylori* infected and non-infected groups, neither at 4 months nor at 7 months (Figs. 6A-B, 7A-B). According to PCoA based on Bray–Curtis distance, there was a tendency for the composition of gut microbiota to differ between male and female mice (Figs. 6E and 7E, Adonis test, p = 0.002, R^2^ = 0.1161). Samples from *H. pylori* infected mice form distinct clusters separate from those of non-infected mice (Adonis test for 4 months, p = 0.008, R^2^ = 0.2444; Adonis test for 7 months, p = 0.001, R^2^ = 0.2331). In parallel with the gastric microbiota, *Helicobacter* showed enrichment in the gut microbiota of infected mice compared to the non-infected group, while *Roseburia* and *Ruminococcfigus* exhibited decreased abundance (Additional file 4: figure S4F).

The gut microbiota was predominantly composed of Bacteroidota and Firmicutes, constituting over 95% of the total. The most abundant genus was a member of *Muribaculaceae*, followed by *Lactobacillus*, *Lachnospiraceae_NK4A136*, and *unclassified_f_Lachnospiraceae*. In the absence of *H. pylori* infection, female mice exhibited higher relative abundance of the beneficial bacteria *Roseburia* and *Ruminococcus,* and a lower abundance of *Streptococcus* compared to male mice (Fig. 6F). After *H. pylori* infection, in comparison with male mice, beneficial *Ruminococcus* and *Bifidobacterium* were enriched in female mice, while *Bilophila, Lachnospiraceae,* and *Romboutsia* were depleted (Figs. 6G, 7G). After 7 months of infection, female mice showed an increase in *Klebsiella* abundance, while male mice exhibited elevated levels of *Escherichia Shigella* compared to the 4 months infection period, highlighting gender disparities in gut microbiota changes (Additional file 5: figure S5B, D).

### Estrogen deprivation resulted in altered composition of gut microbiota following *H. pylori* infection

**Fig. 8:**
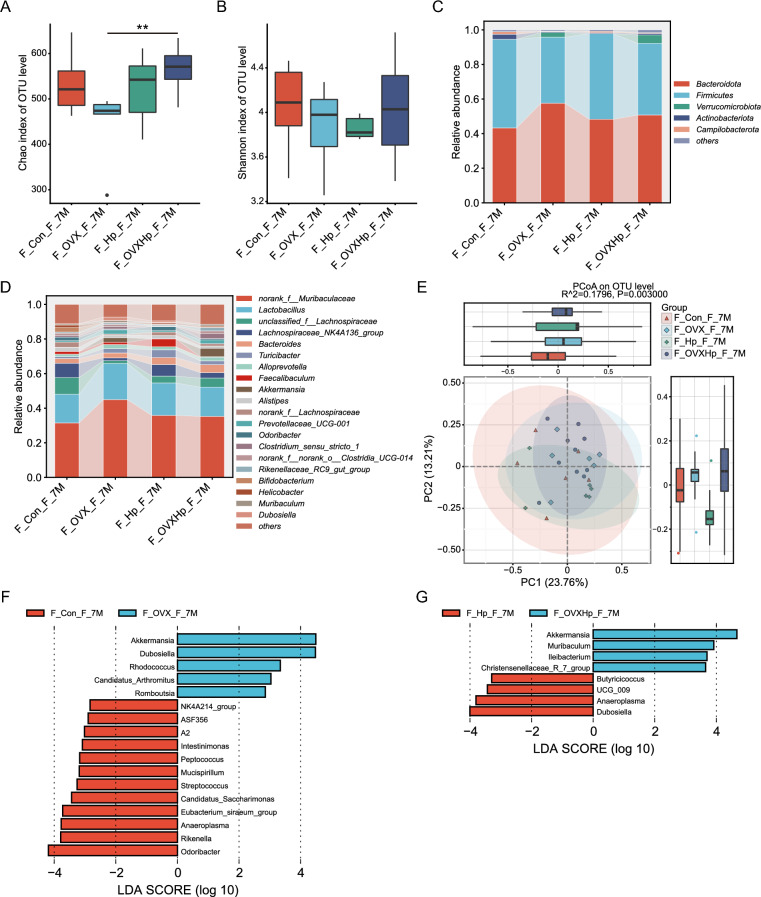
Effect of estrogen deprivation on gut microbiota during *H. pylori* infection. Alpha diversity was calculated by Chao (**A**) and Shannon (**B**) indexes. Percent of community abundance on the phylum (**C**) and genus (**D**) level. **E** Beta diversity analysis of gut microbiota of different groups based on the OTU using the PCoA. **F**, **G** Differences in microbial taxa at the genus level between ovariectomized and intact females were calculated by LEfSe. n = 6–10/group. N represented the number of mice. **p < 0.01. F_Con_F_7M, intact female controls at 7 months. F_OVX_F_7M, ovariectomized females at 7 months. F_Hp_F_7M, intact females after 7 months infection. F_OVXHp_F_7M, ovariectomized females after 7 months infection

Estrogen-induced changes in gut microbiota have been reported to play a crucial role in the sexual dimorphism of various diseases, including metabolic disorders[20]. Estrogen deprivation was induced in female mice through ovariectomy (OVX), with sham surgery performed as the control group before *H. pylori* infection. The analysis of alpha diversity revealed that the Chao index was significantly higher in *H. pylori*-infected mice than in non-infected mice after undergoing ovariectomy (Fig. 8A). No significant difference of microbial diversity and richness was observed between OVX and control groups, irrespective of (Fig. 8A, B). PCoA of Bray–Curtis distance revealed a distinct separation of fecal microbiota between OVX and control mice (Fig. 8E, Adonis test, p = 0.003, R^2^ = 0.1796).

At the phylum level, the relative abundance of Verrucomicrobiota was elevated in OVX mice compared to the controls (Fig. 8C). In the absence of *H. pylori*, OVX mice showed enrichment of *Akkermansia* and *Rhdococcus*, while *Rikenella, Staphylococcus, Odoribacter* were more abundant in the control group (Fig. 8F). The over-representation of *Akkermansia* was consistently observed in OVX mice after *H. pylori* infection, along with an increase in *Staphylococcus* and a decrease in *Butyricicoccus* (Fig. 8G). These results demonstrated that estrogen deprivation induced the dysbiosis of gut microbiota.

### Co-housing ovariectomized and intact female mice normalized gastric lesions induced by *H. pylori* infection

**Fig. 9:**
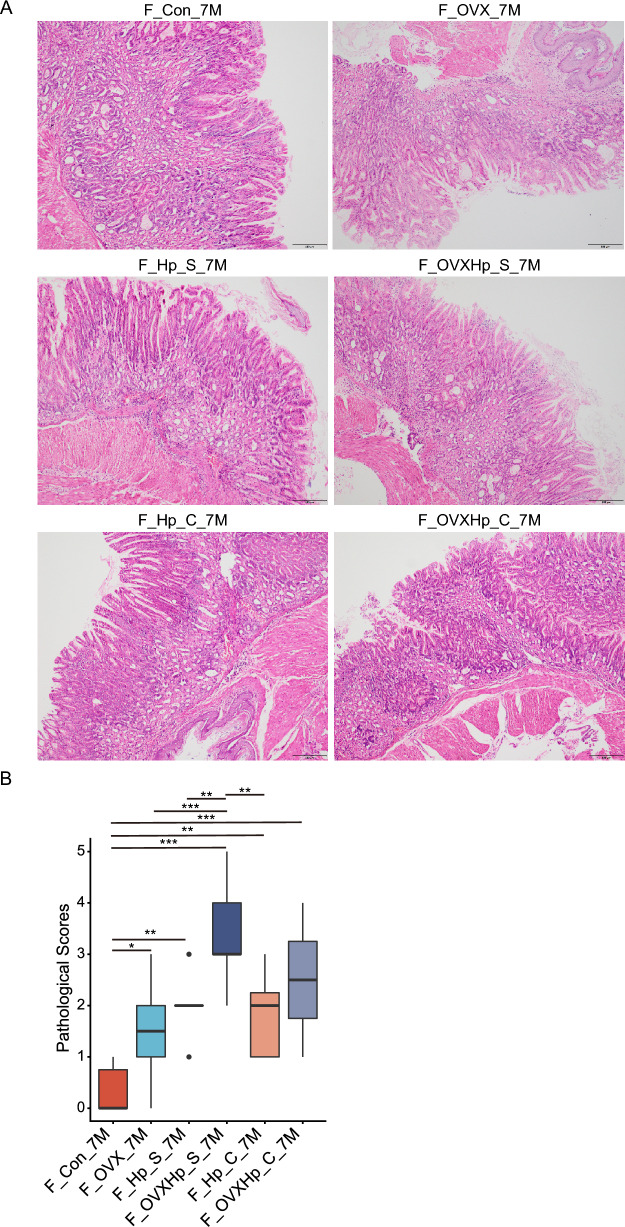
The exacerbation of gastric lesions induced by ovariectomy was alleviated after co-housing. Ovariectomized and intact female mice, both infected with *H. pylori*, were co-housed to examine the effect of gut microbiota exchange on gastric lesions. **A** Representative histological images of H&E staining from mice housed in individual cages and group housing. **B** Gastric histological scores, including inflammation, intestinal metaplasia and dysplasia. n = 6–10/group. N represented the number of mice. F_Con_7M, female controls. F_OVX_7M, ovariectomized females. F_Hp_S_7M, intact females housed separately with 7-month infection. F_OVXHp_S_7M, ovariectomized females housed separately with 7-month infection. F_Hp_C_7M, intact females that co-housed with ovariectomized mice after 7-month infection. F_OVXHp_C_7M, ovariectomized females that co-housed with intact mice after 7-month infection. *p < 0.05. **p < 0.01. ***p < 0.001

Co-house experiment is a common method to explore the effect of gut microbiota transfer on disease phenotype. Thus, we co-housed OVX mice with intact mice together followed by *H. pylori* infection for 7 months. Hematoxylin and eosin (H&E) staining revealed that the histological scores of the infected OVX females were significantly higher than those intact females when they were fed separately. Additionally, the expressions of MPO, F4/80, TNF-α and IL-1β were elevated in OVX females as compared to intact females (Fig. 3A–D). Interestingly, the discrepancy of gastric pathology between *H. pylori*-infected OVX and intact females was notably diminished after their co-housing (Fig. 9A-B). There was no significant difference in the histological scores between co-housed OVX and intact females. These findings suggest that the alterations of gut microbiota induced by estrogen deprivation might involve in the exacerbation of gastric lesions after *H. pylori* infection.

## Discussion

In this study, we found that the gastric lesions were more severe in male mice compared to female mice after *H. pylori* infection. Concurrently, the composition of gastric and gut microbiota differed between male and female mice, with a higher trend of microbial diversity in females compared to males. Interestingly, we noted that the effects of *H. pylori* infection on gastric and gut microbiota exhibited sexual dimorphism. The relative abundance of beneficial *Bifidobacterium* was consistently higher in females compared to males regardless of the infection duration. Furthermore, the gastric injury was aggravated in ovariectomized mice, along with the alterations of gut microbiota. The discrepancy in gastric pathology between ovariectomized and intact females was diminished after co-housing.

Accumulating evidence suggest that *H. pylori* infection could reshape the structure of microbiota in both the stomach and gut, potentially contributing to *H. pylori*-associated diseases. Our previous study found that the gastric microbial diversity was significantly reduced in patients with *H. pylori* infection, with the predominance of *Helicobacter*[21]. Additionally, certain non-*H. pylori* bacteria, specifically oral bacteria, have been implicated in the progression of gastric carcinogenesis[22]. In line with previous studies, we observed that the abundance of some pathogens including *Helicobacter, Streptococcus, Lactobacillus* was strikingly increased and beneficial bacteria such as *Roseburia, Rhodococcus, Ruminococcus*, were decreased after *H. pylori* infection. A recent study identified a strain of *Streptococcus*, known as *Streptococcus anginosus*, as a pathogen that was found to be enriched in patients with gastric cancer and capable of inducing the gastritis-atrophy-metaplasia-dysplasia sequence in mice[23]. Unexpectedly, a large number of studies observed the over-representation of *Lactobacillus* in patients with gastric cancer. Given the probiotic properties of *Lactobacillus* with tumor-suppressive effects, it is possible that its enrichment in gastric cancer is attributed to the alterations of microenvironment during carcinogenesis, including the reduction of gastric acid, which facilitates its colonization[24]. In addition to alterations in the gastric microbiota, individuals infected with *H. pylori* also displayed notable distinctions in the composition of their intestinal microbiota compared to those who are uninfected[25]. Consistently, we also observed a lower abundance of *Ruminococcus*, *Roseburia*, and *Eubacterium*, alongside a higher abundance of *Helicobacter* in fecal samples from *H. pylori*-infected group. Therefore, the infection of *H. pylori* led to disruptions in both gastric and gut microbiota.

In this study, we observed gender-specific differences in the gastric lesions induced by *H. pylori*, with male mice exhibiting higher scores for pathological parameters compared to females. Previous studies have shown that men tend to present with larger, higher-stage gastric cancer than women, as well as having a poorer prognosis[26]. Meanwhile, the gastric lesions induced by *H. pylori* infection developed more rapidly and aggressively in male mice than in females, corroborating our study, though the mechanism remains unclear[27]. Emerging evidence suggests that sex disparities in the gut microbiota may influence gender predisposition to diseases[28]. A number of bacteria have been found to exhibit different abundance between male and female microbiomes[29]. Studies have implicated that elevated estrogen levels correlate with increased abundance of *Bacteroidetes*, reduced levels of *Firmicutes*, and increased diversity[30]. Notably, postmenopausal women with optimal health status exhibit an enrichment of *Bifidobacteria*, which shows a positive correlation with estrogen levels[31]. Another study also demonstrated that supplementation of estrogen can induce shifts in gut microbiota diversity in mice, with an increase in the levels of *Bifidobacteria*[32, 33]. Additionally, the intricate role of gut microbiota in regulating estrogen levels involves the secretion of β-glucuronidase, an enzyme crucial for deconjugating estrogens into their active forms. This process is vital for the enterohepatic circulation and bioavailability of estrogen[34]. Specific bacterial genes encode these enzymes involved in estrogen metabolism. The dysbiosis of gut microbiota could lead to a decrease in the deconjugation of estrogens, resulting in reduced circulating estrogens. This scenario can potentially contribute to the development of conditions such as obesity, metabolic syndrome, and cancer[35].

Our study revealed significant differences in the composition of both gastric and gut microbiota between male and female mice. Furthermore, we observed gender disparities in the alterations of microbiota in response to *H. pylori* infection. Notably, a higher abundance of *Bifidobacterium* was observed in female mice compared to male mice. A previous study has shown that *H. pylori*-infected patients who developed aggressive gastric diseases had a significantly low relative abundance of *Bifidobacterium*[36]. A study involving a Singaporean cohort revealed a significant 62-fold decrease in *Bifidobacterium* abundance in early gastric neoplasia and low-risk intestinal metaplasia[37]. Similarly, a meta-analysis confirmed a diminished presence of *Bifidobacterium* in GC cases[38]. Remarkably low levels of fecal *Bifidobacterium* were noted in GC patients, suggesting potential indirect relationships between *Bifidobacterium* depletion and gastric carcinogenesis[39]. Moreover, the anti-tumor effect of *Bifidobacterium* has been demonstrated both in vitro and in vivo, suggesting its protective role against cancer[40]. Additionally, we observed the enrichment of short chain fatty acids-producing bacteria, including *Faecalibaculum* and *Butyricicoccus*, in female mice compared to males. Microbiota-derived butyrate is capable to reverse the immunosuppressive factors PD-L1 and IL-10 in patients with gastric cancer[39].

A growing number of studies have shown that the abundance of *Escherichia-Shigella* was upregulated in GC cases[41]. These bacteria showed a significant contribution to the development of GC through the production of carcinogenic N-nitroso compounds, which induce over expression of pro-oncogenes, angiogenesis, and inhibition of apoptosis[42]. Additionally, those pathogenic bacteria potentially fuel malignant transformation by producing ROS, which induce DNA damage and perpetuate inflammation[43]. Taken together, our findings suggest that sex-specific changes of gastrointestinal microbiota in response to *H. pylori* infection may be intricately linked to the dichotomy susceptibility to gastric carcinogenesis.

To explore whether estrogen mediates the disparity of *H. pylori*-induced gastric carcinogenesis between genders, we established an ovariectomized mouse model. As anticipated, we observed that ovariectomized mice exhibited more severe gastric lesions compared to their intact counterparts, in line with a previous study[44]. Of note, our findings showed that the composition of gut microbiota in ovariectomized females was significantly different from the intact females, indicating the effect of estrogen on gut microbiota modulation. At the genus level, we found that the relative abundances of *Akkermansia, Muribaculum* were over-represented after estrogen deprivation, while *Butyricicoccus* and *Anaeroplasma* were depleted. The augmentation of *Akkermansia* and *Muribaculum* following ovariectomy has been observed in other studies[45, 46]. Despite being recognized as a next-generation probiotic, *Akkermansia* has been reportedly implicated in the development of colorectal carcinogenesis induced by *H. pylori* infection, attributed to its mucus-degrading capability[47]. Thus, the precise role of *Akkermansia* in gastric carcinogenesis necessitates additional causal validation. Interestingly, we found that the disparity in gastric lesions between ovariectomized and intact mice was diminished after co-housing, indicating the effect of estrogen in modulating gut microbiota to protect against gastric injury. Recent advances have suggested that estrogen, a pivotal regulator of the gut microbiome, modulates the gut microbiome composition by stimulating the proliferation of bacteria that produce short chain fatty acids (SCFAs). These SCFAs play a crucial role in suppressing inflammatory signaling pathways, bolstering gut barrier function and optimizing energy metabolism[48]. The dysbiosis of ovariectomy-associated gut microbiota has been demonstrated to exacerbate gastrointestinal permeability and inflammation, culminating in metabolic dysfunction[20].

There are several limitations to this study. Firstly, the study is disadvantaged by its use of 16S rRNA gene sequencing instead of metagenomics sequencing, which hinders the interpretation of data at the species level and for functional analysis. Second, this study provides evidence of association not causality. Further studies are warranted to explore whether and how female-enriched bacteria, particularly *Bifidobacterium*, contribute to protection against *H. pylori*-associated gastric cancer. Finally, we have solely reported the phenomenon of pathological discrepancies accompanied with the dysbiosis of gastrointestinal microbiota. The specific molecular mechanisms through which the microbiome influences host responses to *H. pylori* associated gastric cancer needs further investigation.

### Perspectives and significance

In summary, we have shown that the structure of gastrointestinal microbiota changed significantly after *H. pylori* infection. Moreover, the alterations of gastrointestinal microbiota triggered by *H. pylori* infection presented distinct patterns between male and female mice, characterized by an increased abundance of beneficial bacteria like *Bifidobacterium* and a decreased abundance of pathogenic bacteria like *Escherichia_Shigella* in female mice. Meanwhile, female mice displayed less severe gastric lesions than males, a phenomenon that was eliminated after estrogen deprivation. Furthermore, we observed that co-housing ovariectomized and intact female mice had a neutralizing effect on their gastric lesions. These findings deepen our understanding of the gender-specific gastrointestinal microbiota, which may contribute to the sex dimorphism observed in gastric carcinogenesis. Therefore, elucidating the role of the gastrointestinal microbiota in the initiation and progression of gastric cancer will offer novel perspectives and a foundational framework for gastric cancer prevention strategies.

## Supplementary Information

**Additional file 1**. **Figure 1** The colonization of *H. pylori* in the stomach of mice. The gastric tissues were harvested from the mice and Giemsa staining has been administered to examine *H. pylori*. Red arrows indicate colonization by *H. pylori* infection (x1000). M_Hp, H. pylori infected males; F_Hp, *H. pylori* infected females; F_OVXHp, ovariectomized female mice infected with H. pylori; M_Con, non-infected males; F_Con, non-infected females.

**Additional file 2**. **Figure 2** Effects of *H. pylori* infection on the gastric microbiota of INS-GAS mice. Alpha diversity indexes, including Chao (**A**) and Shannon (**B**), were calculated. An overview of microbial composition at the phylum (**C**) and genus (**D**) level. **E** Beta diversity analysis utilizing principal coordinates analysis based on Bray-Curtis distance. **F** Differential genera were identified using LEfSe analysis. Con, control mice. Hp, *H. pylori* infected mice.

**Additional file 3**. **Figure 3** Chronological alterations of gastric microbiota in males and females. The LEfSe analysis was conducted to identify the differentially abundant bacteria between the 4-month and 7-month groups in males (**C**,** D**) and females (**A**,** B**) with and without H. pylori infection. F_Con_G_4M, female controls at 4 months. F_Hp_G_4M, females following 4 months infection. M_Con_G_4M, male controls at 4 months. M_Hp_G_4M, males following 4 months infection. F_Con_G_7M, female controls at 7 months. F_Hp_G_7M, females following 7 months infection. M_Con_G_7M, male controls at 7 months. M_Hp_G_7M, males following 7 months infection.

**Additional file 4**. **Figure 4** Impact of *H. pylori* infection on the gut microbiota of INS-GAS mice. **A**–**B**) Alpha diversity of gut microbiota from mice with and without *H. pylori* infection. Relative abundance of bacterial phyla (**C**) and genera (**D**). **E** PCoA of the Bray-Curtis distances for infected and non-infected mice. (F) Differences in microbial taxa at genus level after *H. pylori* infection were shown by LEfSe. Con, control mice. Hp, *H. pylori* infected mice.

**Additional file 5**. **Figure S5** Chronological changes of gut microbiota in males and females. Gut genera with differential abundance between the 4-month and 7-month groups were identified using LEfSe. F_Con_F_4M, female controls at 4 months. F_Hp_F_4M, females following 4 months infection. M_Con_F_4M, male controls at 4 months. M_Hp_F_4M, males following 4 months infection. F_Con_F_7M, female controls at 7 months. F_Hp_F_7M, females following 7 months infection. M_Con_F_7M, male controls at 7 months. M_Hp_F_7M, males following 7 months infection.

## Data Availability

The raw sequencing data of gut microbiota have been uploaded to the NCBI Sequence Read Archive (SRA, http://www.ncbi.nlm.nih.gov/sra) under accession number PRJNA1151686.
