# Supplementary figures and images for: Sex-specific effects of gastrointestinal microbiome disruptions on Helicobacter pylori-induced gastric carcinogenesis in INS-GAS mice
Source: Biol Sex Differ. 2025 Feb 21;16:15. doi: 10.1186/s13293-025-00700-z (PMC11846230; doi:10.1186/s13293-025-00700-z)

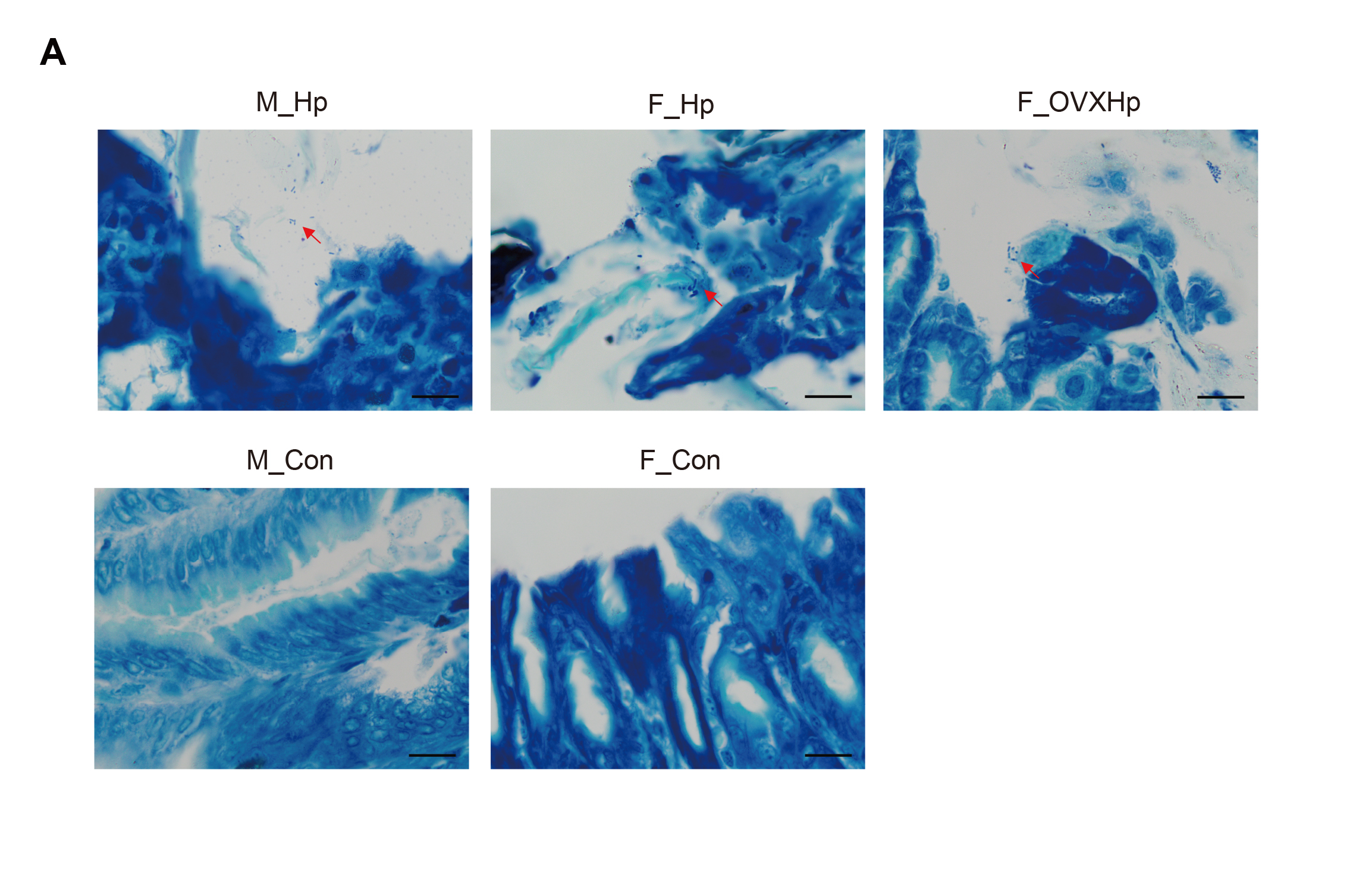

Supplement: Supplementary file 1 — Additional file 1. Figure 1 The colonization of H. pylori in the stomach of mice. The gastric tissues were harvested from the mice and Giemsa staining has been administered to examine H. pylori. Red arrows indicate colonization by H. pylori infection (x1000). M_Hp, H. pylori infected males; F_Hp, H. pylori infected females; F_OVXHp, ovariectomized female mice infected with H. pylori; M_Con, non-infected males; F_Con, non-infected females. [file 13293_2025_700_MOESM1_ESM.jpg]

A

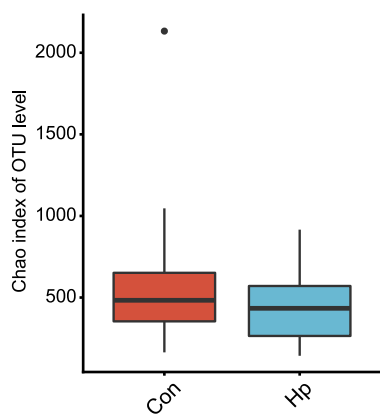

B

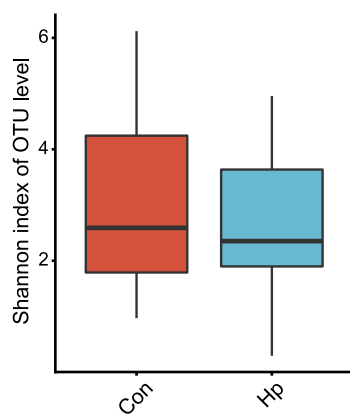

C

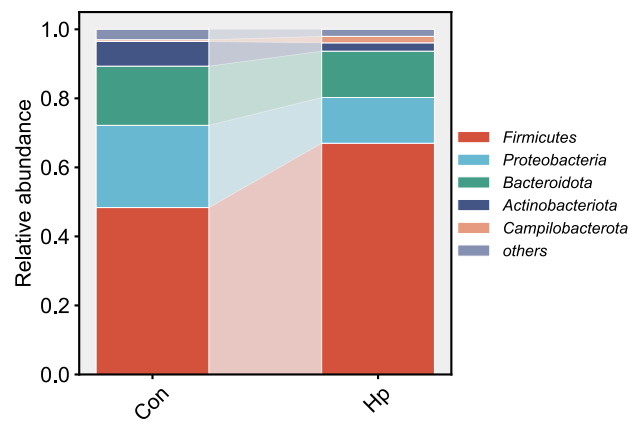

D

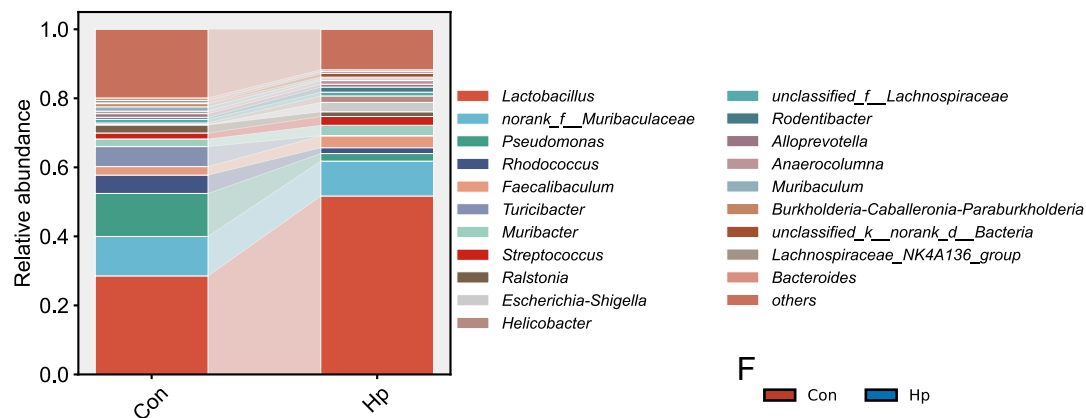

E

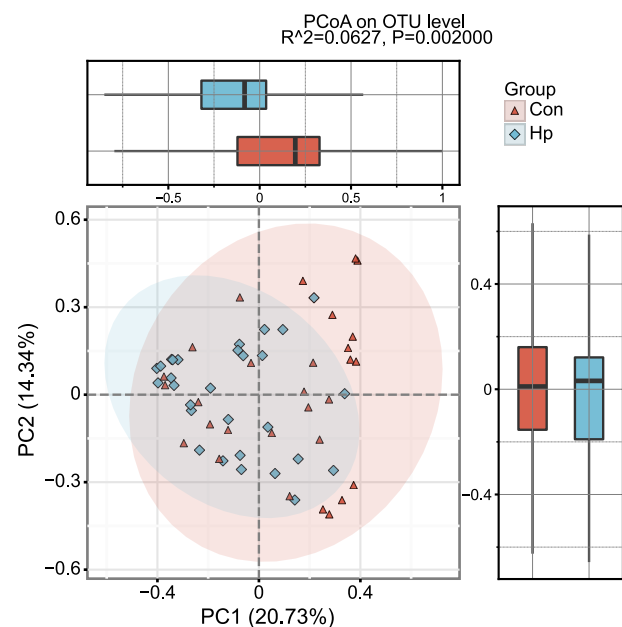

F

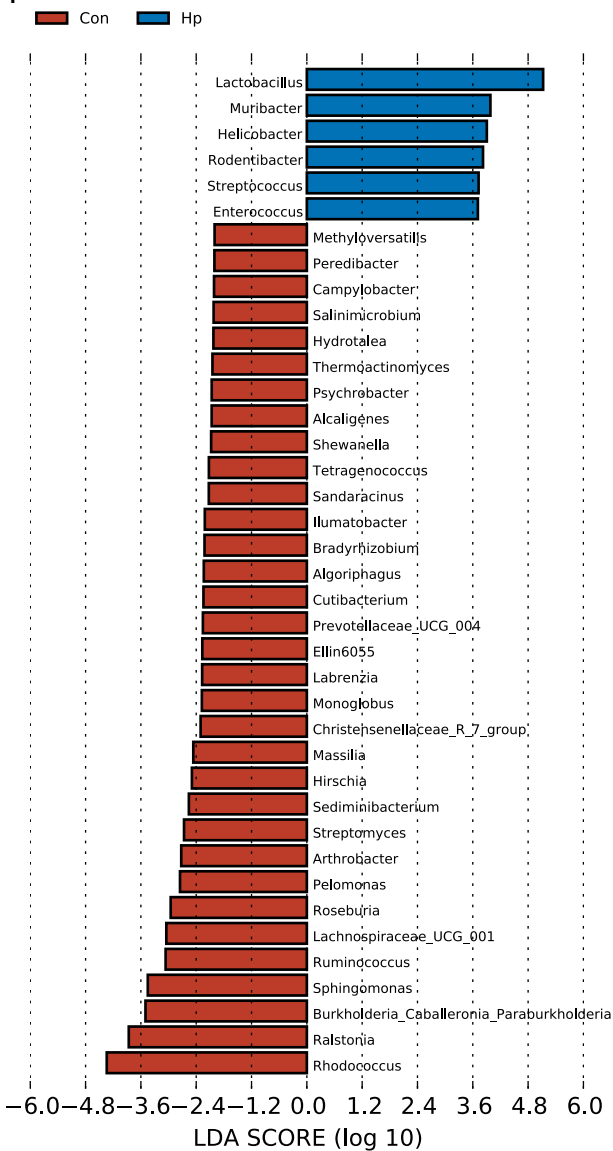

Supplement: Supplementary file 2 — Additional file 2. Figure 2 Effects of H. pylori infection on the gastric microbiota of INS-GAS mice. Alpha diversity indexes, including Chao (A) and Shannon (B), were calculated. An overview of microbial composition at the phylum (C) and genus (D) level. E Beta diversity analysis utilizing principal coordinates analysis based on Bray-Curtis distance. F Differential genera were identified using LEfSe analysis. Con, control mice. Hp, H. pylori infected mice. [file 13293_2025_700_MOESM2_ESM.pdf]

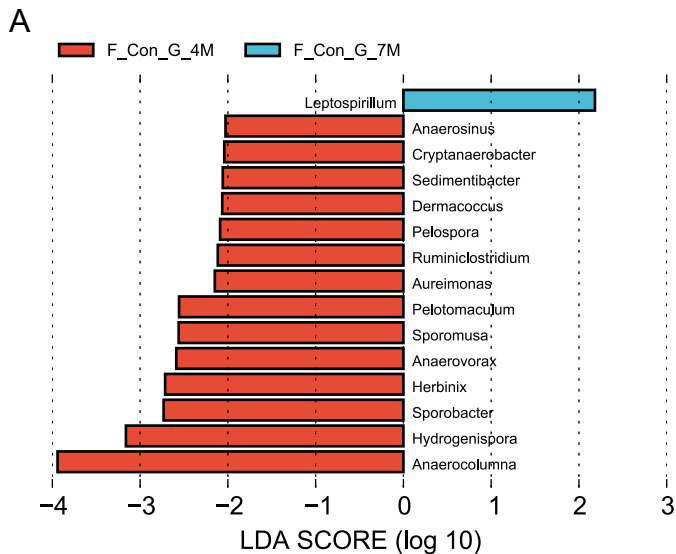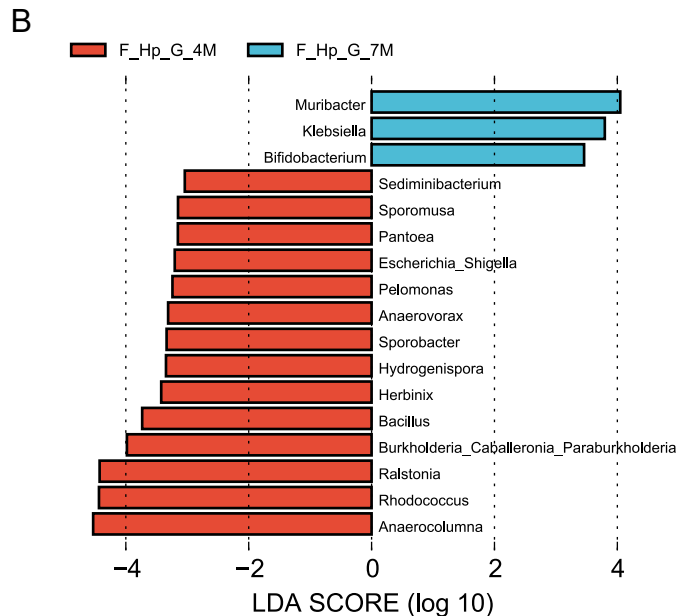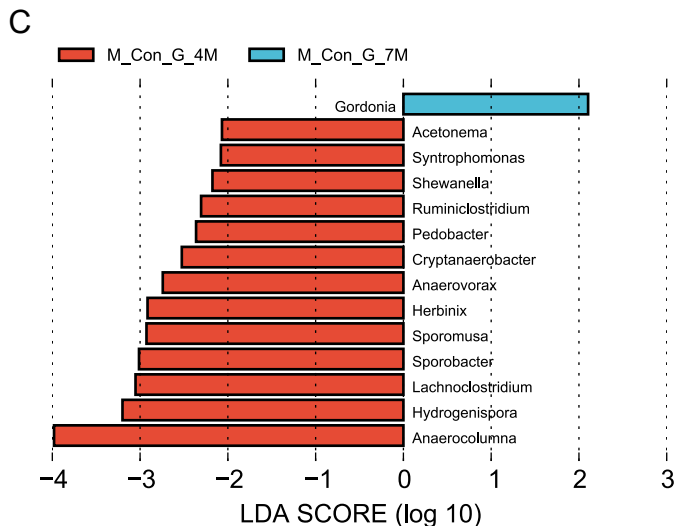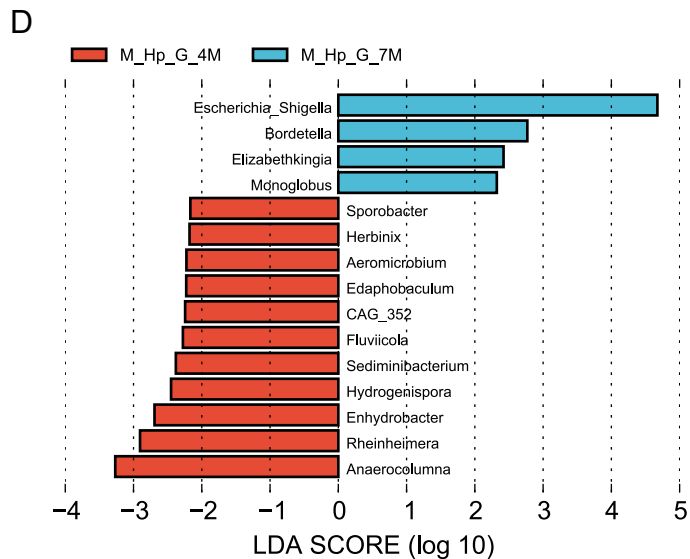

Supplement: Supplementary file 3 — Additional file 3. Figure 3 Chronological alterations of gastric microbiota in males and females. The LEfSe analysis was conducted to identify the differentially abundant bacteria between the 4-month and 7-month groups in males (C, D) and females (A, B) with and without H. pylori infection. F_Con_G_4M, female controls at 4 months. F_Hp_G_4M, females following 4 months infection. M_Con_G_4M, male controls at 4 months. M_Hp_G_4M, males following 4 months infection. F_Con_G_7M, female controls at 7 months. F_Hp_G_7M, females following 7 months infection. M_Con_G_7M, male controls at 7 months. M_Hp_G_7M, males following 7 months infection. [file 13293_2025_700_MOESM3_ESM.pdf]

A

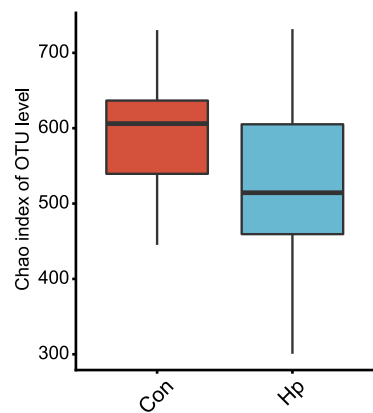

B

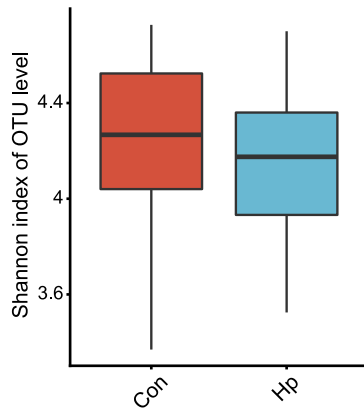

C

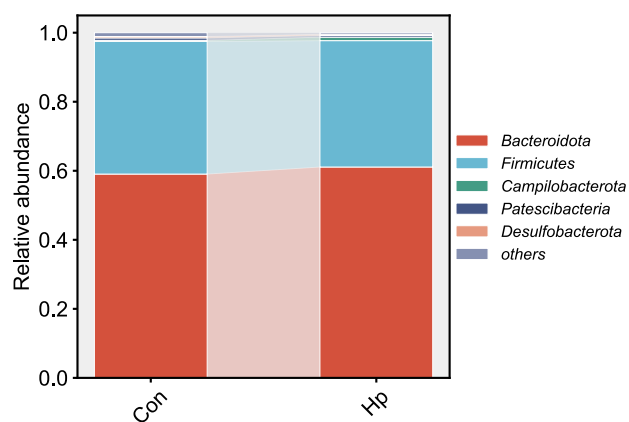

D

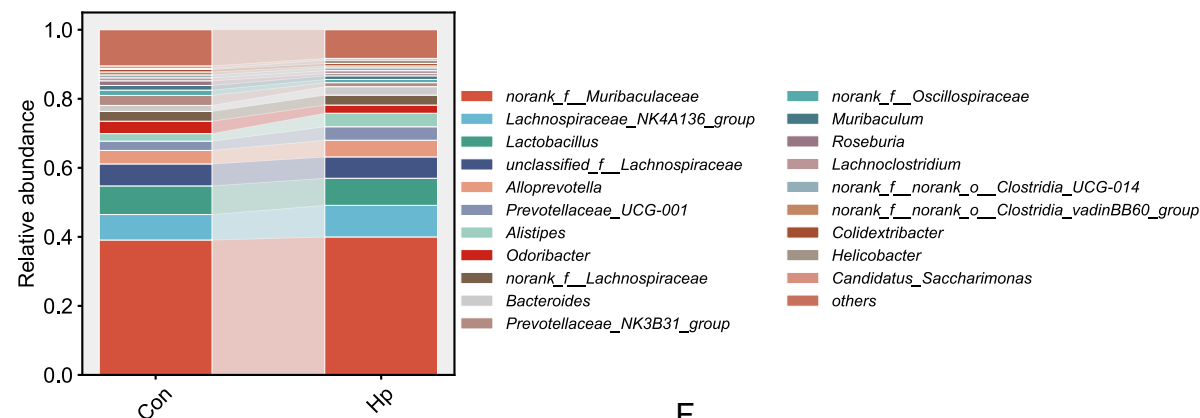

E

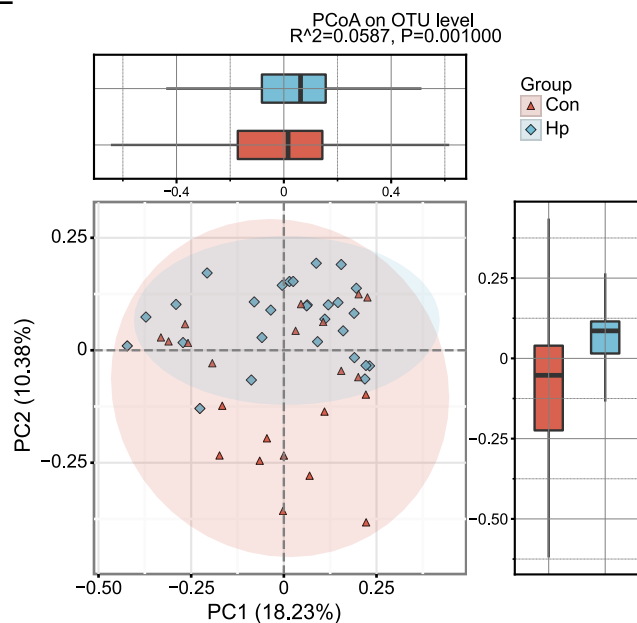

F

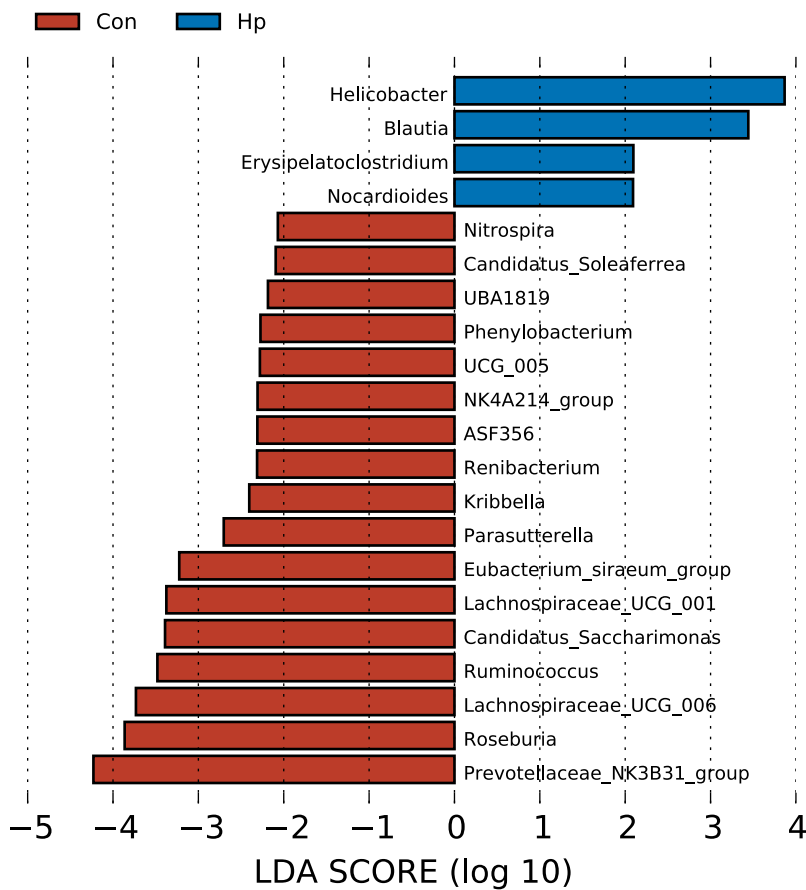

Supplement: Supplementary file 4 — Additional file 4. Figure 4 Impact of H. pylori infection on the gut microbiota of INS-GAS mice. A–B) Alpha diversity of gut microbiota from mice with and without H. pylori infection. Relative abundance of bacterial phyla (C) and genera (D). E PCoA of the Bray-Curtis distances for infected and non-infected mice. (F) Differences in microbial taxa at genus level after H. pylori infection were shown by LEfSe. Con, control mice. Hp, H. pylori infected mice. [file 13293_2025_700_MOESM4_ESM.pdf]

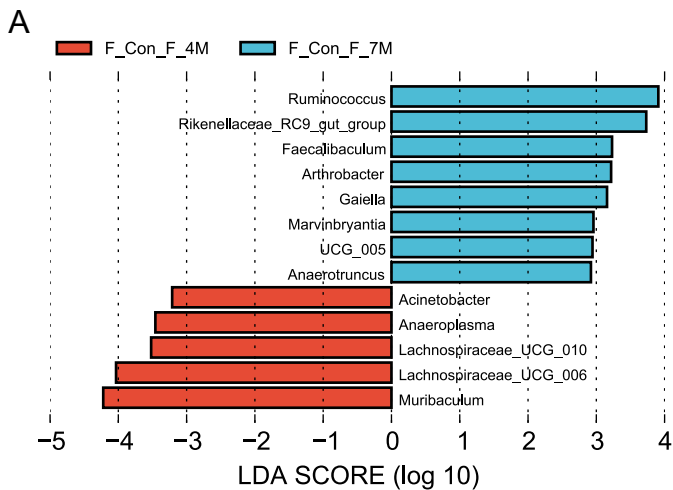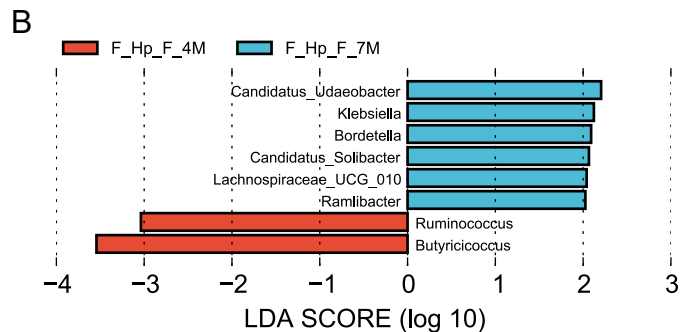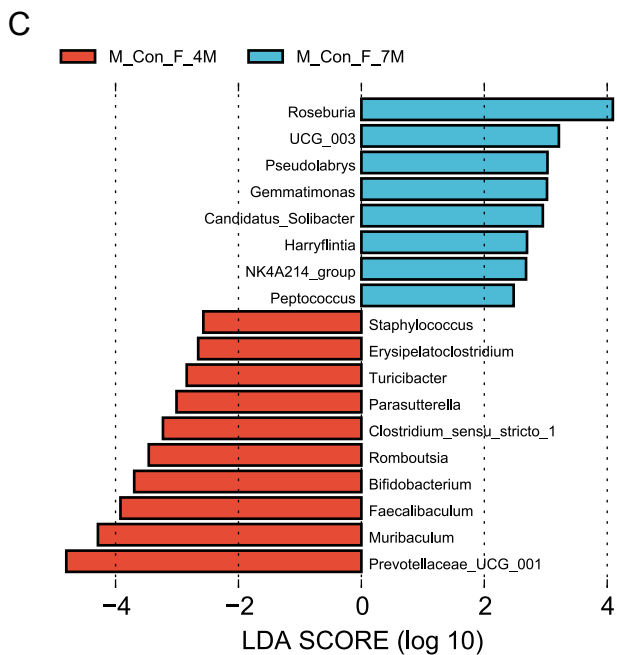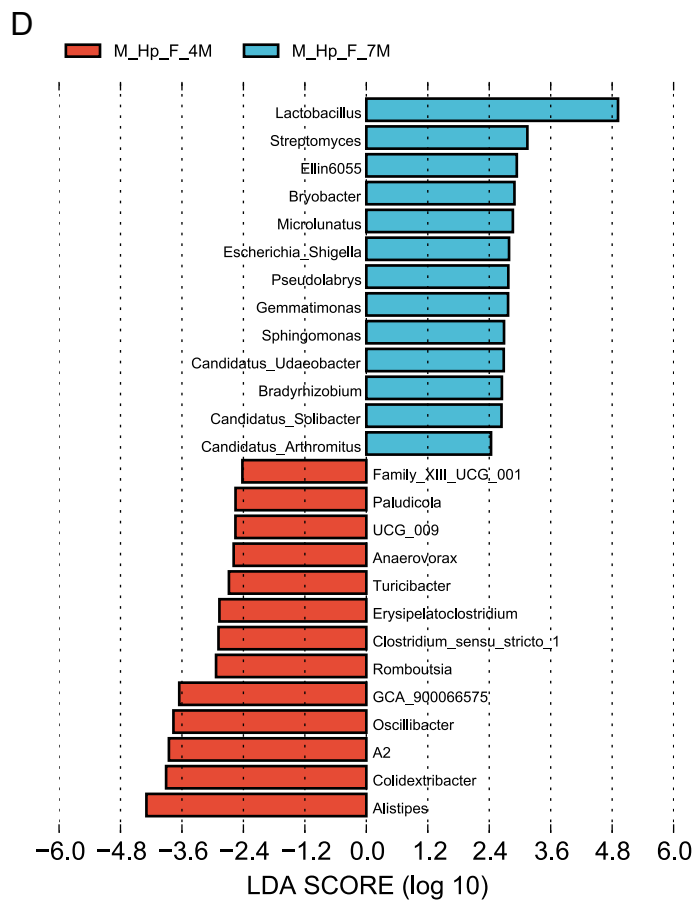

Supplement: Supplementary file 5 — Additional file 5. Figure S5 Chronological changes of gut microbiota in males and females. Gut genera with differential abundance between the 4-month and 7-month groups were identified using LEfSe. F_Con_F_4M, female controls at 4 months. F_Hp_F_4M, females following 4 months infection. M_Con_F_4M, male controls at 4 months. M_Hp_F_4M, males following 4 months infection. F_Con_F_7M, female controls at 7 months. F_Hp_F_7M, females following 7 months infection. M_Con_F_7M, male controls at 7 months. M_Hp_F_7M, males following 7 months infection. [file 13293_2025_700_MOESM5_ESM.pdf]
